# Undetectable plasma viral load predicts normal survival in HIV-2-infected people in a West African village

**DOI:** 10.1186/1742-4690-7-46

**Published:** 2010-05-19

**Authors:** Maarten F Schim van der Loeff, Natasha Larke, Steve Kaye, Neil Berry, Koya Ariyoshi, Abraham Alabi, Carla van Tienen, Aleksandra Leligdowicz, Ramu Sarge-Njie, Zacharias da Silva, Assan Jaye, Dominique Ricard, Tim Vincent, Sarah Rowland Jones, Peter Aaby, Shabbar Jaffar, Hilton Whittle

**Affiliations:** 1 MRC Laboratories Fajara, P.O. Box 273, Banjul, The Gambia; 2 Department of Epidemiology and Population Health, London School of Hygiene & Tropical Medicine, Keppel Street, London WC1E 7HT, UK; 3 Division of Retrovirology, National Institute of Biological Standards and Control/HPA, South Mimms, UK; 4 Department of Clinical Medicine, Institute of Tropical Medicine, Nagasaki University, Nagasaki, Japan; 5 Bandim Health Project, Indepth network, Bissau, Guinea-Bissau

## Abstract

**Background:**

There have been no previous studies of the long-term survival and temporal changes in plasma viral load among HIV-2 infected subjects.

**Methods:**

133 HIV-2 infected and 158 HIV-uninfected subjects from a rural area in North-west Guinea-Bissau, West Africa were enrolled into a prospective cohort study in 1991 and followed-up to mid-2009. Data were collected on four occasions during that period on HIV antibodies, CD4% and HIV-2 plasma viral load.

**Results:**

Median age (interquartile range [IQR]) of HIV-2 infected subjects at time of enrollment was 47 (36, 60) years, similar to that of HIV-uninfected control subjects, 49 (38, 62) (p = 0.4). Median (IQR) plasma viral load and CD4 percentage were 347 (50, 4,300) copies/ml and 29 (22, 35) respectively.

Overall loss to follow-up to assess vital status was small, at 6.7% and 6.3% for HIV-2 infected and uninfected subjects respectively. An additional 17 (12.8%) and 16 (10.1%) of HIV-2 infected and uninfected subjects respectively were censored during follow-up due to infection with HIV-1. The mortality rate per 100 person-years (95% CI) was 4.5 (3.6, 5.8) among HIV-2 infected subjects compared to 2.1 (1.6, 2.9) among HIV-uninfected (age-sex adjusted rate ratio 1.9 (1.3, 2.8, p < 0.001) representing a 2-fold excess mortality rate associated with HIV-2 infection.

Viral load measurements were available for 98%, 78%, 77% and 61% HIV-2 infected subjects who were alive and had not become super-infected with HIV-1, in 1991, 1996, 2003 and 2006 respectively. Median plasma viral load (RNA copies per ml) (IQR) did not change significantly over time, being 150 (50, 1,554; n = 77) in 1996, 203 (50, 2,837; n = 47) in 2003 and 171 (50, 497; n = 31) in 2006. Thirty seven percent of HIV-2 subjects had undetectable viraemia (<100 copies/ml) at baseline: strikingly, mortality in this group was similar to that of the general population.

**Conclusions:**

A substantial proportion of HIV-2 infected subjects in this cohort have stable plasma viral load, and those with an undetectable viral load (37%) at study entry had a normal survival rate. However, the sequential laboratory findings need to be interpreted with caution given the number of individuals who could not be re-examined.

## Background

The sooty mangabey simian immunodeficiency virus (SIV), the ancestor of HIV-2, is estimated to have crossed from monkey to man around 1940, resulting in an outbreak of HIV-2 subtype A in West Africa [1]. HIV-2 has remained endemic in West Africa, and now in this region both HIV-2 and HIV-1 infections are prevalent, providing an opportunity to draw comparisons between the natural history and immunopathogenesis of the two viruses [2].

A prevalence of HIV-2 of 8-10% has been recorded in some settings [3], but is now thought to be stable or falling across West Africa [4]. Median survival of HIV-1 infected subjects in sub-Saharan Africa in the absence of antiretroviral therapy is about 10 years [5,6], similar to that in developed countries, and plasma viral load and CD4 count have been identified as strong markers of prognosis [7,8]. Because of the paucity of community-based HIV-2 cohorts, median survival with HIV-2 has not been widely documented, but survival with HIV-2 was longer than that with HIV-1 in a hospital-based study in Gambia, especially at higher CD4 count [9,10]. Similarly, in an urban community-based study among individuals more than 35 years old [11], 9-year HIV-2-associated mortality was only twice that of HIV-uninfected subjects [12]. The long-term survival of HIV-2 infected subjects is not known.

A number of studies have shown that HIV-2 infections are associated with lower plasma viral load [13], slower CD4 decline [14,15] and a lower incidence of AIDS [16] than HIV-1 in the same study populations. Also, CD4 count and plasma viral load in HIV-2 are predictors of mortality [17-20]. However, some of this evidence is from hospital-based studies which contained subjects with more advanced disease with a relatively short follow-up period. Whether these markers predict survival over the long-term in the community or the clinic is not known.

We have conducted a community-based cohort study of HIV-2 infected people in rural West Africa followed from 1991 through to 2009. Here we report changes in plasma viral load and survival over this 18-year follow-up, which is the longest on record and one of the few with laboratory variables.

## Methods

### Study area and population

The study was conducted in Caió, a village in north-western Guinea-Bissau, West Africa. The study comprised a population of about 10,000 individuals, mostly subsistence farmers. The sex ratio is unbalanced because many men migrate for work. Women may also leave in search for work in the region's urban centres.

A serological survey conducted in the community between 1989-1991 showed HIV-2 prevalence among adults aged ≥ 15 years to be 8% (240 subjects), peaking at 19% in men aged 45-54 years, and 17% in women aged 35-44 years [21]. HIV-2 infected subjects and an equal number of HIV-seronegative controls, broadly matched for age and sex, were visited at home by field staff. Those who were present at home and gave informed consent were invited to participate in the study [22]. Field staff were unaware of the HIV status of the study subjects.

Subjects were followed-up annually to record vital status (data collected up until mid-2009). They were also invited for a clinical examination and asked for a blood sample in 1991, 1996, 2003 and 2006. Blood samples were tested for HIV status, and those HIV-infected were tested for CD4% and plasma viral load.

All subjects received pre-test counselling before HIV testing during each of the four study rounds. Post-test counselling and HIV test results were available for those who wanted to know their results. Subjects had access to a physician-led clinic and were offered free medical treatment, including that for anaemia, malaria, and syphilis, according to national guidelines. Prophylaxis against opportunistic infections with co-trimoxazole was offered from 1999 onwards to HIV infected subjects who were aware of their diagnosis and who were either symptomatic or had a CD4% <28%. Antiretroviral treatment was not available in Guinea-Bissau at the time of the field study, but is now in place.

### Laboratory methods

The laboratory methods of the 1991 and 1996 study rounds are described by Wilkins et al. [21], Ricard et al. [22] and Berry et al. [20]; methods for 2003 and 2006 are described in Leligdowicz et al. [23]. In brief, for measuring HIV-2 plasma viral loads in 2003 and 2006, RNA was extracted from 200 μl plasma using a silica gel purification method, and an aliquot of the extract equivalent to 40 μl of the original sample was amplified in a single-tube reverse transcribed-PCR (Qiagen "One-Step", Hilden, Germany). A standard curve was generated using RNA extracted from cell culture supernatants of HIV-2 (strain CBL-23). Each reaction was spiked with approximately 100 copies of an internal control. This control was a 1 kb RNA molecular construct spanning the PCR primer binding sites and replacing the probe binding site with a 25 base randomised sequence. The control was extracted and co-amplified with the test sample and probed separately. Results were calculated as signal ratios of test sample to internal control and copy numbers determined by comparison with the standard curve. A positive control was included in every assay run to control for inter-assay variation. The assay had a dynamic range of 100 to 1,000,000 RNA copies/ml of plasma. Samples with undetectable virus were assigned a value half that of the detection threshold of the test, for the purposes of analysis.

Twenty-nine samples of HIV-2 infected subjects taken in 1996 were re-assayed with the current method. The mean virus loads were 3.81 log_10 _and 3.96 log_10 _copies/ml by old and current method respectively. Fifteen (52%) were less than 0.5 log_10 _different, and 25 (86%) were within 1 log_10 _difference; the agreement was acceptable [beta = 0.75 (95%CI 0.48, 1.02); r^2 ^= 0.55].

We chose to analyse the CD4 data by percentage rather than by absolute count. In the early years of the epidemic, due to the lack of automated counting machines, we were obliged to estimate lymphocyte counts by manual methods which introduced considerable observer variation and error. Moreover a batch of slides from Caio destined for lymphocyte counting was damaged during transit on bad roads. Thus in addition to providing a more robust measurement, the use of CD4 percentage obtained by FACS analysis allowed us to use the full data set at baseline.

### Ethics

This study was approved by the MRC Laboratories/Gambia Government Joint Ethics Committee, the London School of Hygiene & Tropical Medicine Ethics Committee, and the Research Committee of the Ministry of Health of Guinea-Bissau.

### Statistical methods

Continuous data were presented as medians and interquartile ranges (IQR), since these data were non-normally distributed. Continuous data were compared between groups using the Wilcoxon rank sum or Kruskal-Wallis test. Categorical data were compared using the chi-squared test. Correlation was assessed using Spearman's correlation coefficient. Mortality rates were calculated using Poisson regression with time calculated from enrolment in 1991 to either the date of death or the end of the study in mid-2009 or the last date seen alive for those lost-to-follow-up (i.e. permanently moved away from the village or were not re-identified). HIV-2 infected subjects who also seroconverted to HIV-1 in a subsequent survey were censored from the date on which they were known to be infected with HIV-1. Controls who seroconverted to either HIV-1 or HIV-2 were censored likewise. Analyses were conducted using Stata 10 (Stata Corp, College Station, TX, USA).

## Results

### Baseline characteristics

In 1991, 133 HIV-2 infected and 158 HIV-uninfected subjects were enrolled. Almost all were from the Manjago ethnic group (Table 1). Median age (Interquartile range [IQR]) of HIV-2 infected subjects was 47 (36, 60) compared with 49 (38, 62) of HIV-uninfected subjects (p = 0.4). The male: female ratio was similar among HIV-2 infected and HIV-uninfected subjects (p = 0.4). Among HIV-2 infected subjects median (IQR) plasma viral load and CD4 percentage were 347 (50, 4,300) copies per ml and 29 (22, 35). Forty eight of the 130 subjects (37%) who were tested had an undetectable level of virus (<100 copies/ml), whereas only 22 (17%) had levels above 10,000 copies/ml. The median viral load was lower among women (137, IQR 50, 127,000) compared to men (755, IQR 125, 286,000). Plasma viral load was associated inversely with CD4 percentage (r = -0.30, p < 0.001) but not with age (r = 0.07, p = 0.4).

**Table 1:** Baseline characteristics in 1991.

	HIV-2 infected (N = 133)	Uninfected (N = 158)
***Age in years ***^***a***^		
<30	13 (10%)	29 (18%)
30-39	24 (18%)	27 (17%)
40-49	39 (30%)	31 (20%)
50-59	20 (15%)	32 (20%)
60-69	28 (21%)	20 (13%)
≥ 70	8 (6%)	19 (12%)
***Sex***		
Males	42 (32%)	43 (27%)
***Ethnicity***^***b***^		
Manjago	125 (94%)	152 (96%)
Other	7 (5%)	6 (4%)

^a ^Data missing for one HIV-2 infected subject

^b ^Data missing for one HIV-2 infected subject

### Follow-up and survival of HIV-2 and HIV-uninfected subjects

**Figure 1:**
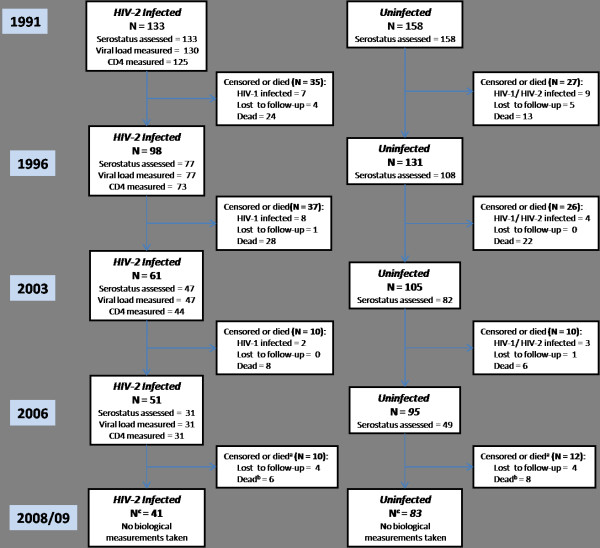
**Flowchart showing follow-up status of subjects with respect to biological measurements and survival over the course of the study**. At some time points subjects were known to be alive e.g. they were seen at later time points, but measurements were not taken from them. ^a ^After 2006 biological measurements were not taken and so data on seroconversions not available between 2006 and 2009. ^b ^Subjects who had died by mid-2009. ^c ^Known to be alive at latest census in 2009.

Subjects were followed-up annually through to mid-2009, with blood samples collected in 1991, 1996, 2003 and 2006 (Figure 1). Loss to follow-up with regard to vital status was small, being 6.7% and 6.3% respectively for the infected and uninfected subjects respectively. In addition a further 17 (12.8%) HIV-2 infected and 16 (10.1%) uninfected subjects became infected with HIV-1 during follow-up. The proportion of HIV-2 infected and HIV-uninfected subjects known to be alive in 1996, 2003, 2006 and 2008/9 and who did not seroconvert to HIV-1 were 74% and 83%, 46% and 66%, 38% and 60%, and 31% and 53% respectively. The median (IQR) follow-up time was 11.8 (5.3, 17.3) years among HIV-2 infected subjects and 17.7 (9.5, 18.6) among HIV-uninfected subjects (p < 0.001).

**Figure 2:**
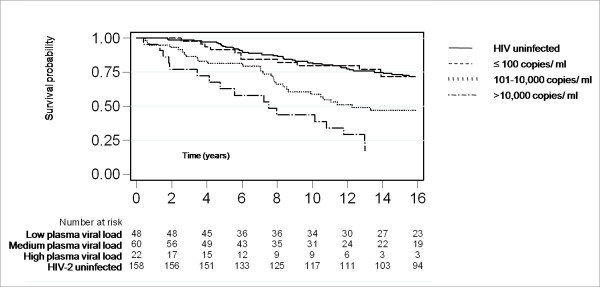
**Kaplan-Meier survival of HIV-2 infected (stratified by baseline plasma viral load) and HIV-uninfected subjects**.

Baseline characteristics of those followed-up successfully through to 2008/09, those who died and those lost to follow-up or censored are shown in Table 2. The distribution of age, sex, percentage CD4 and plasma viral load of those alive were similar to those lost to follow-up or censored, but differed substantially from those who died. The overall mortality rate per 100 person-years (95% CI) was 4.5 (3.6, 5.8) among HIV-2 infected subjects compared to 2.1 (1.6, 2.9) among the HIV-uninfected population. Figure 2 shows the Kaplan-Meier survival of HIV-2 and HIV-uninfected subjects. Mortality increased with age and was higher among men than women, but this was true for both HIV-infected and HIV-uninfected subjects (Table 3). Thus the relative difference or rate ratio decreased with age. Among subjects 60 years or older in 1991, there was no significant difference in survival between HIV-2 and HIV-uninfected. Overall, the mortality rate ratio (95% CI) adjusted for age category and sex was 1.9 (1.3, 2.8, p < 0.001). Mortality rates for both groups were marginally higher during 1999 to 2009 compared to the earlier time period (Table 3). The mortality of women compared with men was 0.42 (95% CI 0.24, 0.74) among HIV-uninfected subjects after adjusting for age category and 0.51 (95% CI 0.31, 84) among HIV-2-infected.

**Table 2:** Baseline characteristics (in 1991) of subjects according to HIV diagnosis and follow-up status in 2008/09.

	HIV-2 infected			HIV-uninfected		
	
	Alive in mid 2009 (N = 41)	Died by mid 2009 (N = 66)	Lost or censored by mid 2009 (N = 26)	Alive in mid 2009 (N = 83)	Died by mid 2009 (N = 49)	Lost or censored by mid 2009 (N = 26)
***Sex, number (%)***						
Male	12 (29)	27 (64)	3 (7)	18 (42)	20 (47)	5 (12)
Female	29 (32)	39 (43)	23 (25)	65 (57)	29 (25)	21 (18)
***Age in years, median (IQR)***	45 (33, 55)	56 (42, 66)	43 (37, 49)	41 (28, 52)	63 (51, 71)	42 (37, 53)
***CD4 percentage, median (IQR)***	32 (25, 40)	25 (18, 32)	32 (26, 35)			
***Plasma viral load copies per ml, median (IQR)***	79 (50, 775)	1630 (127, 12,225)	90 (50, 530)			

**Table 3:** Crude mortality rates by age and sex among HIV-2 - infected and HIV uninfected individuals.

	HIV-2 infected		HIV-uninfected		
		
	Number died/total per category (%)	Mortality rate per 100 person-years (95% CI)	Number died/total per category (%)	Mortality rate per 100 person-years (95% CI)	Rate ratio (95% CI)
***Age group***^***a, b***^					
<40	15/37 (41)	3.4 (2.0, 5.6)	7/56 (13)	0.8 (0.4, 1.7)	4.2 (1.7, 10.2)
40-59	23/59 (39)	3.4 (2.3, 5.1)	15/63 (24)	1.6 (1.0, 2.7)	2.1 (1.1, 4.1)
≥ 60	28/36 (78)	8.5 (5.9, 12.3)	27/39 (69)	5.8 (4.0, 8.5)	1.5 (0.9, 2.5)
***Sex***					
Male	27/42 (64)	6.1 (4.2, 8.8)	20/43 (46)	3.7 (2.4, 5.8)	1.6 (0.9, 2.9)
Female	39/91 (43)	3.9 (2.8, 5.3)	29/115 (25)	1.7 (1.2, 2.4)	2.3 (1.4, 3.7)
***Calendar time***					
1991-1998	37/133 (28)	4.3 (3.1, 6.0)	24/158 (15)	1.9 (1.3, 2.8)	2.3 (1.4, 3.8)
1999-2009	29/84 (35)	4.8 (3.4, 7.0)	25/120 (21)	2.5 (1.7, 3.7)	1.9 (1.1, 3.3)
***Overall***	66/133 (50)	4.5 (3.6, 5.8)	49/158 (31)	2.2 (1.6, 2.9)	2.1 (1.5, 3.0)

^a ^Age at enrolment

^b^Missing data for one individual

### Association between baseline CD4 percentage, plasma viral load and the mortality of HIV-2 infected subjects

In the univariable analysis both plasma viral load and CD4 percentage were associated independently with mortality (Table 4). The association with plasma viral load was very strong (Figure 2). Mortality in those with undetectable viral load was similar to that of the uninfected subjects, whereas those with a viral load of 10,000 copies/ml or greater had a rate of 10.4 per 100 years, which is similar to that for HIV-1 in the region [11].

**Table 4:** Association between CD4 percentage, plasma viral load measured in 1991 and mortality over a 18 year follow-up among HIV-2 infected subjects.

			Univariable		Multivariable ^a^	
			
	Number	Mortality rate per 100 person-years (95% CI)	Rate ratio (95% CI)		Rate ratio (95% CI)	P value
***Plasma viral load ***^***b***^***, copies per ml***						
≤ 100	15/48 (31)	2.4 (1.4, 4.0)	1.0	<0.001	1	<0.001
101-10,000	31/60 (52)	4.9 (3.5, 7.0)	2.0 (1.1, 3.8)		2.0 (1.0, 4.2)	
>10,000	18/22 (82)	10.4 (6.6, 16.5)	4.3 (2.2, 8.6)		5.6 (2.5, 12.5)	
***CD4 percentage ***^***c***^						
≥ 28%	26/70 (37)	3.1 (2.1, 4.6)	1.0	0.007	1	0.2
<28%	34/55 (62)	6.3 (4.5, 8.8)	2.0 (1.2, 3.3)		1.4 (0.8, 2.5)	

^a ^Adjusted for age category, sex, CD4 percentage and plasma viral load

^b ^Missing data for 3 individuals

^c ^Missing data for 8 individuals

In a multivariable analysis with age category, sex, plasma viral load category and CD4 percentage category, the association between mortality and plasma viral load became stronger. The adjusted rate ratio for the medium viral load category (101-10,000 copies/ml) was 2.0 (95%CI 1.0, 4.2) and 5.6 (95%CI 2.5,12.5) for the high viral load category ( > 10,000 copies/ml). When plasma viral load was analysed on a continuum, the adjusted mortality ratio was 1.8 (95%CI 1.4, 2.3; p < 0.0001) for every log_10 _increase in viral load.

In the multivariable analysis, there was no evidence of an association between CD4 percentage and mortality. The adjusted rate ratio for those with a CD4 percentage <28% compared with ≥ 28% was 1.4 (95% C I 0.8, 2.5; p = 0.2). When CD4 percentage was analysed on a continuum, the adjusted mortality rate ratio was 1.3 (95% C I 0.98,1.7: p = 0.07) for every absolute 10% decrease in CD4.

### Changes in HIV-2 plasma viral load and CD4% over time

Table 5 shows the plasma viral load and CD4% among HIV-2 infected subjects during follow-up. The proportion of HIV-2 infected subjects who were alive and had not been censored due to loss to follow-up or HIV-1 seroconversion, from whom viral load was available in 1991, 1996, 2003, and 2006 were 98%, 78%, 77% and 61% respectively; the corresponding proportions for CD4 measurements were 94%, 74%, 72% and 61%. The median plasma viral load of those tested did not differ significantly between the time points (p = 0.5) whereas the median CD4% showed a small increase over time (p = 0.01). Plasma viral load measured in 1996, 2003 and 2006 was correlated with baseline (1996: r = 0.68, p < 0.001, n = 76; 2003: r = 0.53, p < 0.001, n = 47; 2006: r = 0.38, p = 0.04, n = 31), although this correlation decreased over time.

**Table 5:** Changes in HIV-2 plasma viral load and CD4 percentage over time.

	1991 (N = 133)	1996 (N = 97)	2003 (N = 47)	2006 (N = 31)
***Plasma viral load, copies ml***				
Median (IQR)^a^	348 (50, 4,300)	150 (50, 1,554)	203 (50, 2,837)	171 (50, 497)
Median change (IQR)^b^	N/A	0 (-140, 825)	0 (0, 1,534)	0 (-2, 373)
Category, number (%)				
<100	48 (36)	33 (42)	20 (43)	13 (42)
100-9,999	60 (45)	32 (41)	19(40)	16 (52)
≥ 10,000	22 (17)	12 (15)	8 (17)	2 (6)
Not tested	3 (2)	1 (1)	0 (0)	0 (0)
***CD4+ (% of lymphocytes)***				
Median (IQR)^c^	29 (22, 35)	32 (26, 38)	32 (26, 37)	33 (27, 43)
Median change (IQR)^d^	N/A	3 (-4, 8)	3 (-1, 8)	5 (-2, 9)
Category				
≥ 28%	70 (53)	48 (61)	28 (60)	22 (71)
<28%	55 (41)	25 (32)	16 (34)	9 (29)
Not tested	8 (6)	5 (6)	3 (6)	0 (0)

^a ^Data were available for 130 subjects in 1991, 77 in 1996, 47 in 2003 and 38 in 2006

^b ^Median change compared to previous time point. Data were available at both time points for 76 subjects in 1996, 42 in 2003 and 26 in 2006

^c ^Data were available for 125 subjects in 1991, 73 in 1996, 44 in 2003 and 31 in 2006

^d ^Median change compared to previous time point. Data were available at both time points for 69 subjects in 1996, 31 in 2003 and 26 in 2006

Changes in plasma viral load over time were also analysed according to follow-up status (died, lost to follow-up or censored, and alive). The median change between 1991 and 2003 among those alive in 2006 was 0 (IQR -57, 657 copies per ml; n = 27), and this was similar to those who were lost to follow-up or censored (0, IQR -632, 1082 copies per ml; n = 12) (p = 0.8). The median change between 1991 and 2003 for those who died after 2003 was 20,236 (IQR -780, 57,328 copies per ml; n= 6) (p = 0.1).

Thirty-one subjects had plasma viral load measurements, and 30 were tested for CD4% in both 1991 and 2006. Their median (IQR) age at baseline was 46 (41, 57) years. The median (IQR) plasma viral load was 105 (50, 335) and 171 (50, 497) copies per ml respectively and median (IQR) CD4 percentage 29% (25, 35) and 33% (27, 42) respectively. Fifteen (48%) had undetectable plasma viral load in 1991, of whom 13 (42%) had maintained an undetectable viral load 15 years later in 2006.

## Discussion

The disease course and pathogenicity of HIV-2 infections are recognised to differ from HIV-1, although detailed descriptions of long-term survival with HIV-2 over prolonged periods have not been fully documented. In this unique 18-year community-based study conducted in rural Guinea -Bissau, we have demonstrated the mortality of HIV-2 infected subjects to be approximately twice that of HIV-uninfected subjects. This confirms findings from studies which had shorter durations of follow-up [9,10,12,20,22] and extends the earlier observations of this cohort [20]. Mortality rate ratios of HIV-2-infected and HIV-uninfected decreased with age (at enrolment). In fact the rate ratios diminished with time either because background mortality of the elderly is high or because many of the HIV-2 infected old people were long term non-progressors with a normal lifespan. Mortality, after adjusting for age, was lower in women than men who also have higher plasma viral load. In contrast mortality in rural Uganda was increased 10 fold in HIV-1 infected subjects and was similar for men and women. In the Ugandan study, those over the age of 55 years died more rapidly than younger patients or their age matched uninfected counterparts [6].

Ascertainment of vital status was good in this close-knit rural West African community, with a total loss to follow up of only 6.5%. However, follow up sampling for laboratory tests was less satisfactory as, apart from those who died, many subjects were not in the village at the time of the surveys or were identified to have seroconverted to HIV-1, so were censored. Thus, the longitudinal aspects of the laboratory studies need to be interpreted with some caution as the outcomes of those not retested are not known (though their baseline characteristics were broadly similar to those who were re-sampled). In addition we do not know precisely when the subjects in our cohort were infected with HIV-2, but even if infection was acquired recently and the death rate was high among those lost to follow-up, our study confirms that many HIV-2 infected subjects may have a long-lifespan.

Baseline plasma viral load among our HIV-2 cohort was generally very low as previously reported [20]. Plasma viral load was undetectable in 37% of the subjects who had a normal lifespan; this is in sharp contrast to almost all community-based HIV-1 studies in Africa. For example, in a study among pregnant women in Gambia only 3% of HIV-1 infected subjects had an undetectable viral load, and the median viral load was 30-40 fold higher [24]. In the much smaller proportion (17%) of HIV-2 infected subjects in Caio with a high viral load (>10,000 copies per ml) these had greatly decreased survival, as has been found in HIV-1 infection in Bissau and The Gambia [11,18].

Both HIV-2 plasma viral load and CD4 percentage predicted survival. The association with HIV-2 plasma viral load was very strong; and in multivariable analysis mortality among those with plasma viral load exceeding 10,000 HIV-2 RNA copies per ml was 5 fold higher than in those with undetectable plasma viral load, whose mortality rate was not appreciably different from that in HIV-2-uninfected subjects. The association between CD4 percentage and survival was weaker and lost significance after adjusting for age, sex and plasma viral load. Our results confirm those of previous studies showing lower viral replication in HIV-2, which suggested that HIV-2 plasma viral load may predict prognosis [13,17-20]. We are unable to say with certainty how plasma viral load changed over time in those who died since our sampling was not done with sufficient frequency. However, in the small number tested there was a large but statistically non-significant rise in plasma viral load (median increase 20,000 copies/ml) in the 12 year period before their death.

Importantly, and for the first time, we have shown that amongst the survivors who were followed-up successfully and did not seroconvert to HIV-1, plasma viral load did not change appreciably over the 18 years of follow-up. Thus, there is a substantial proportion of individuals in HIV-2 infection in whom viral load remains set and stable at a very low level over decades, compared to the much higher set points typically described for HIV-1 [25]. Understanding the biological significance of this observation seems key to unravelling differences in the enhanced survival of the majority of HIV-2 subjects compared to HIV-1. Both host genetic and viral factors are likely to be important. The same HIV-2 subjects with a low or undetectable viral load and a normal CD4 percentage have been shown to have strong T-cell responses to the HIV-2 gag protein, particularly directed towards the highly conserved region represented by peptide 46 in the Major Homology Region [23,26]. The findings raise the intriguing possibility of developing a therapeutic vaccine targeted at the gag epitopes identified in this study for the treatment of those with progressive disease. As viral load is already well controlled in the majority of subjects, the chances of success may be higher than in HIV-1 infection.

However, there remains a small proportion of subjects where host control of virus infection has failed, in those with a high viral load and a mortality rate similar to those with HIV-1 infection. An analogous situation has been described in wild chimpanzees infected with an SIV chimpanzee strain (SIVcpz), thought to have evolved after the transmission of a SIV from red-capped sooty mangabey monkeys [27], where a higher viral load also correlates with mortality. Current research based on the cohort suggests that high viral load is associated with HLA B*1503 subtype which is common in the Manjago ethnic group and confined to populations in sub-Saharan Africa [28]. A previous study of viral genotype in Caio village in which sequencing was limited to a small proportion of the genome, suggested that viral genotype determined disease progression and that virulence factors are multiple and scattered through out the genome [29]. A recent analysis of HIV-2 p26 capsid variation in plasma viral sequences rescued from this cohort has shown that a proline in positions 119, 159 and178 in the capsid protein predicts a low viral load. Conversely if other amino acids are occupying these positions the viral load is high [30]. The study raises interesting questions as to how the low-replicating proline mutants are maintained in the population, as transmission is strongly related to viral load.

## Conclusion

This unique field study of HIV-2 infection in a rural community demonstrates the power of coupling good epidemiological data with detailed laboratory investigations. Divergent patterns of viral pathogenicity have resulted in two distinct clinical outcomes which provide a great opportunity to examine the key elements of host protection and viral virulence. Further studies need to be informed by complete sequencing of host and viral genomes and a deeper understanding of their phenotypic interactions. Such basic knowledge is sorely needed to understand correlates of immunity and their effect on the evolution of the virus, which will inform the design of HIV vaccines which to date have met with little success.

## Competing interests

The authors declare that they have no competing interests.

## Authors' contributions

MS was responsible for field work and wrote the first draft of the paper. NL did the statistical analysis. SK, NB, KA, AA, AL, RSN, ZdS and AJ, and SRJ were responsible for the laboratory analyses. TV, AL, CvT and PA organised and co-ordinated the follow-up of subjects. SJ wrote subsequent drafts of the paper. DR and HW designed the original study with help from PA. HW gave overall direction to the project and wrote the last draft with help from SRJ All authors commented on drafts of the paper.
